# Retraction: miR‐655 suppresses epithelial‐to‐mesenchymal transition by targeting Prrx1 in triple‐negative breast cancer

**DOI:** 10.1111/jcmm.18314

**Published:** 2024-04-24

## Abstract

Zhi‐Dong Lv, Bin Kong, Xiang‐Ping Liu, Li‐Ying Jin, Qian Dong, Fu‐Nian Li, Hai‐Bo Wang, miR‐655 suppresses epithelial‐to‐mesenchymal transition by targeting Prrx1 in triple‐negative breast cancer. *Journal of Cellular and Molecular Medicine*, 20: 864–873. https://doi.org/10.1111/jcmm.12770

The above article, published online on 28 January 2016 in Wiley Online Library (wileyonlinelibrary.com), has been retracted by agreement between the authors, the journal Editor‐in‐Chief, Stefan Constantinescu, The Foundation for Cellular and Molecular Medicine, and John Wiley and Sons Ltd. In 2022, the authors asked to retract the article as they found that some images were mistakenly duplicated from their previously published papers. Subsequently, an investigation into concerns raised by a third party revealed inappropriate duplications and manipulation of images between this (Figures 2B,D, 3A, 5B, 6C,D and 7B) and five other articles (with the same first or corresponding author) that were published previously or in the same year in a different scientific context. Due to the number and the level of errors identified in the published figures, the editors consider the conclusions of this manuscript substantially compromised. Although the authors initially asked for the retraction, they did not agree to the retraction wording.

Zhi‐Dong Lv, Bin Kong, Xiang‐Ping Liu, Li‐Ying Jin, Qian Dong, Fu‐Nian Li, Hai‐Bo Wang, miR‐655 suppresses epithelial‐to‐mesenchymal transition by targeting Prrx1 in triple‐negative breast cancer. *Journal of Cellular and Molecular Medicine*, 20: 864–873. https://doi.org/10.1111/jcmm.12770

The above article, published online on 28 January 2016 in Wiley Online Library (wileyonlinelibrary.com), has been retracted by agreement between the authors, the journal Editor‐in‐Chief, Stefan Constantinescu, The Foundation for Cellular and Molecular Medicine, and John Wiley and Sons Ltd. In 2022, the authors asked to retract the article as they found that some images were mistakenly duplicated from their previously published papers. Subsequently, an investigation into concerns raised by a third party revealed inappropriate duplications and manipulation of images between this (Figures 2B,D, 3A, 5B, 6C,D and 7B) and five other articles (with the same first or corresponding author) that were published previously or in the same year in a different scientific context. Due to the number and the level of errors identified in the published figures, the editors consider the conclusions of this manuscript substantially compromised. Although the authors initially asked for the retraction, they did not agree to the retraction wording.

